# Defective Drainage

**Published:** 1874-02

**Authors:** 


DEFECTIVE DRAINAGE.—At the last meeting of the State Medical Society the Committee on Hygiene presented an interesting report which provoked an instructive discussion, confined chiefly to the subject of defective drainage. A full report of the discussion and of the report of the committee is contained in the *Sanitarian* for March, and will amply repay a careful study. The report is accompanied by maps of a portion of New York, and of Brooklyn and Kings County. The subject of drainage is one of vast importance, and the neglect of proper attention thereto will be followed by an increased amount of sickness and death. We shall in our next present our readers with a valuable paper on The Diffusion of Typhoid Fever by Means of Drinking Water, from the pen of Prof. Austin Flint, which will contain some valuable remarks upon this subject.

